# Antibodies on demand: a fast method for the production of human scFvs with minimal amounts of antigen

**DOI:** 10.1186/1472-6750-11-61

**Published:** 2011-06-02

**Authors:** Ingrid Babel, Rodrigo Barderas, Alberto Peláez-García, J Ignacio Casal

**Affiliations:** 1 Functional Proteomics Laboratory. Centro de Investigaciones Biológicas (CIB-CSIC). Ramiro de Maeztu 9, Madrid 28040, Spain

**Keywords:** scFv antibodies, *in vitro *protein expression, phage display, antibody microarrays

## Abstract

**Background:**

Antibodies constitute a powerful tool to study protein function, protein localization and protein-protein interactions, as well as for diagnostic and therapeutic purposes. High-throughput antibody development requires faster methodologies with lower antigen consumption.

**Results:**

Here, we describe a novel methodology to select human monoclonal recombinant antibodies by combining *in vitro *protein expression, phage display antibody libraries and antibody microarrays. The application of this combination of methodologies permitted us to generate human single-chain variable fragments (scFvs) against two proteins: green fluorescent protein (GFP) and thioredoxin (Trx) in a short time, using as low as 5 μg of purified protein. These scFvs showed specific reactivity against their respective targets and worked well by ELISA and western blot. The scFvs were able to recognise as low as 31 ng of protein of their respective targets by western blot.

**Conclusion:**

This work describes a novel and miniaturized methodology to obtain human monoclonal recombinant antibodies against any target in a shorter time than other methodologies using only 5 μg of protein. The protocol could be easily adapted to a high-throughput procedure for antibody production.

## Background

A crucial challenge of the proteome era is to use the genome information for a better understanding of protein expression, protein cellular distribution and functionality discovery not only in normal but also in pathological processes [1,2]. Antibody development against every human protein is a prerequisite to improve this knowledge. Several high-throughput alternatives have been developed to generate antibodies to the entire proteome [3-5]. The Human Protein Atlas initiative (http://www.proteinatlas.org/) [3,4], the Sanger Institute Antibody Atlas Database, the NCI Clinical Proteomics [5], the HUPO human antibody initiative (http://www.hupo.org/research/hai/) [6], and several EU-funded consortia (ProteomeBinders, AffinityProteome, Affinomics [7-9]; http://www.proteomebinders.org) are all good examples of these alternatives.

The production of mAbs and/or rabbit antibodies requires large amounts of antigens, it is time-consuming due to the immunization step of the animals and, in the case of mAbs, the screening and clone selection can take from 6 months to 1 year [10].The development of recombinant antibodies in single-chain Fv (scFv) formats is a good alternative to obtain high-affinity antibodies against any target without time-consuming immunization [11-14]. The affinity of scFvs for their targets might be comparable to that of mAbs or pAbs and in some cases even higher [15]. As a general rule, scFvs possess several advantages in comparison to IgG or Fabs such as higher tissue penetrance and more rapid clarification [16,17]. Moreover, antibody phage display, M13-based human libraries, is becoming particularly useful for the production and development of antibodies for immunotherapy in different diseases [18-21]. *In vitro *phage display pipelines have been setup to generate antibodies to the complete human proteome, but the selections are still carried out manually [8,9,22]. Screening of phage display antibody libraries is constrained by the necessity of having considerable amounts of antigen, at least 0.1-0.5 mg of protein for the whole procedure (selection, screening and validation).

The necessity of having significant amounts of the purified target protein, not only for production and selection but also for the screening of antibodies, is one of the main problems to develop antibodies, and constitutes a major bottleneck associated to all three alternatives above described [10]. Despite progress in automation, protein expression is a limiting step to get toxic, difficult-to-express or membrane proteins. Rapid, efficient, and cost-effective protein expression and purification strategies are required for the production of antibodies against any target, trying to minimize at the same time, the amount of required protein.

Cell-free expression is a powerful and flexible technology. New advances in this technology have faced the higher demand for high-throughput protein synthesis. These advances include the use of cell-extracts from different backgrounds (prokaryotic or eukaryotic), modulation of the reducing environment for the correct production of disulfide bonds, incorporation of detergents, lipid bilayers or other non-lipoprotein particles for the expression of membrane proteins and, finally, the automation of the procedure [23-26]. Furthermore, cell-free systems offer several advantages over traditional cell-based expression methods, which include lower sensitivity to product toxicity and suitability for high-throughput strategies, because of reduced reaction volumes and processing time. Recent improvements in translation efficiency have resulted in yields comparable to cell-based expression systems for difficult-to-express proteins [27-30].

Bacterial, wheat germ and reticulocyte lysates have been used as *in vitro *expression systems in a wide variety of strategies [31]. Rapid Translation System (RTS) [32], a bacterial-based commercially available cell-free protein expression system, has been used for the high-throughput expression of inner and outer membrane proteins from *Anaplasma marginale *[33]. In 2000, the Riken Structural Genomics Initiative (Japan) reported the bacterial based cell-free protein expression production of about 25% of randomly chosen mouse cDNA clones with yields higher than 0.1 mg/ml [34]. Since then, several developments have significantly improved the yield of *in vitro *expressed proteins in the range of milligrams of protein per mL of reaction mix [27,28,35,36]. Another interesting initiative is the "human protein factory", for the expression of human proteins using the wheat-germ *in vitro *protein expression system and Gateway technology [24]. The authors reported a 97% success rate of protein expression over 13364 human proteins. Among them, they detected soluble proteins in 12682 out of 13364 clones [24]. There have been other successful high-throughput initiatives based on *in vitro *protein expression, with yields up to 6 mg/mL, which have been applied for protein arrays, nuclear magnetic resonance and crystallization studies [37-39].

Regarding the use of low amounts of protein, the microarray format is particularly useful for low consumption and automation. Antibody arrays were initially designed to capture and detect simultaneously multiple analytes with high affinity and selectivity in human biological samples (plasma, serum, tissue ...) in order to study variations between biological statuses [5]. We have explored the use of this proteomic technique for testing scFv antibody libraries against different antigens at the same time in order to identify specific scFvs, increasing the throughput of the screening step with minimal requirements of protein. In this report, we propose a novel methodology to develop scFv antibodies using human phage display antibody libraries in a short time (no more than four weeks) with only 5 μg of protein. Furthermore, this method could be automated in a high-throughput format to obtain "antibodies on demand" against any target, taking advantage of cell-free protein expression, antibody phage display and scFv antibody microarrays.

## Results

### Production of human scFvs against cell-free expressed antigens

**Figure 1:**
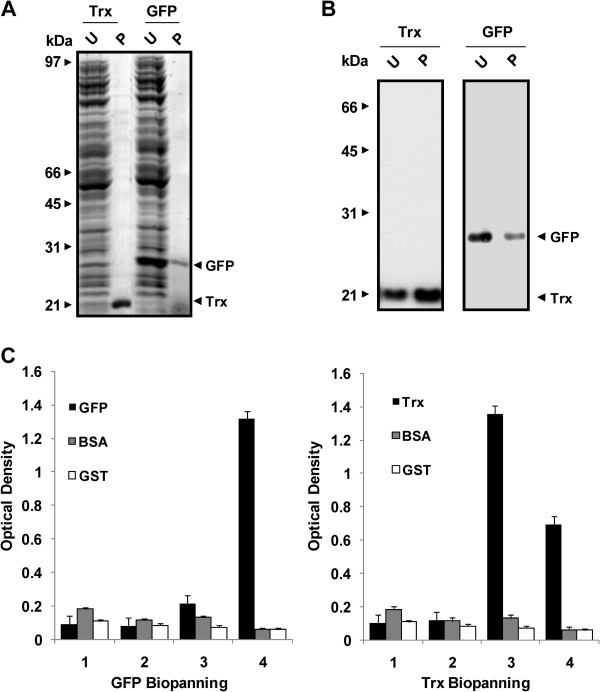
**Cell-free protein expression and characterization**. A) Cell free GFP and Trx expression and purification by TALON™ Dynabeads^® ^was assessed by SDS-PAGE and Coomassie Blue staining. U, unpurified total protein extract. P, protein purified with TALON™ Dynabeads^®^. B) Western blot analysis of the proteins by using peroxidase-labeled anti-His. U, unpurified total protein extract. P, Proteins purified with TALON™ Dynabeads^®^. C) ELISA characterization of the polyclonal phages after different rounds of biopanning. A peroxidase-labeled anti-M13 was followed by TMB incubation to develop the signal. His-tagged BSA and GST were used as negative controls.

The cDNAs-encoding GFP and Trx were cloned into pIVEX and pET32b plasmids, respectively, and used for the *in vitro *transcription/translation reactions. The cell-free expression yielded between 5-10 μg of purified protein per 50 μL RTS reaction. The purity and homogeneity of the expressed proteins were confirmed by SDS-PAGE and western blot using 10 μl of RTS reaction (Figure 1A, B).

Proteins were immobilized on the TALON™ magnetic beads and used to get antibodies by phage display. After extensive washing with PBS, TALON Dynabeads^® ^were divided in five tubes containing 10 μL of beads, one for each of the four rounds of selection with the libraries and the fifth one for Alexa Fluor 647-protein labelling. A remarkable phage enrichment for both proteins was observed by ELISA after 3 or 4 rounds of biopanning (Figure 1C).

### scFv antibody microarray preparation

**Figure 2:**
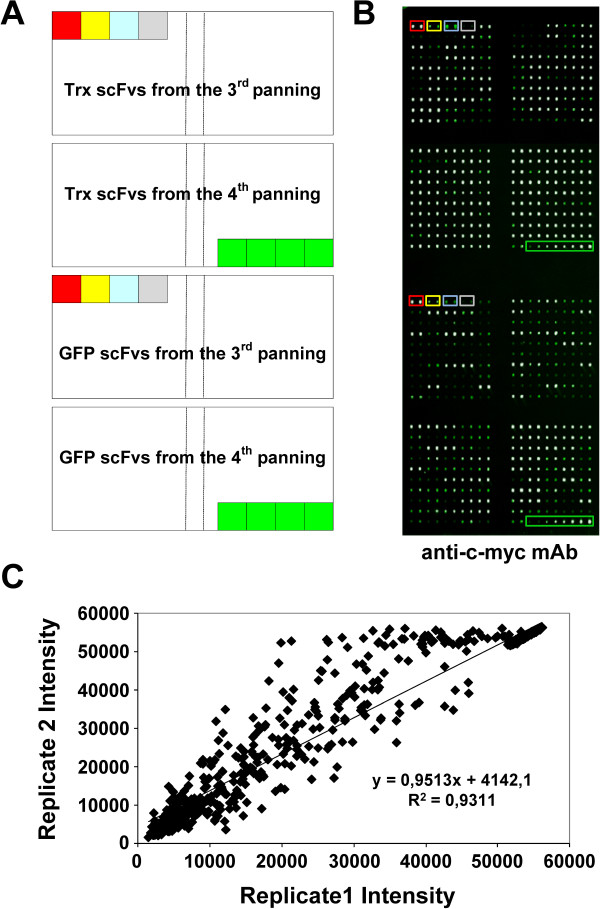
**Printing conditions and scFv antibody microarrays reproducibility**. A) Printing map of a microarray comprising 384 scFvs against GFP or Trx. Controls used in the assay were: red box, mAb anti-T7Tag, 1:10 diluted. Yellow box, mAb anti-T7Tag 1:100 diluted. Blue box, crude Trx (top) or GFP (bottom) RTS extract 1:10 diluted. Grey box, printing buffer. Green box, TA4 anti-gastrin17 scFv; from right to left 1:10, 1:100, 1:1000 and 1:1000 dilutions. B) A representative image of a microarray probed with an anti-c-myc antibody to assess the correct printing of the scFvs. For detecting c-myc antibody, slides were incubated with Alexa Fluor 555-labeled goat anti-mouse IgG antibodies. White spots indicate a saturation of the green signal intensity. C) scFvs were spotted in duplicate onto FAST nitrocellulose coated slides to verify the intra-assay reproducibility. Replicated spots showed a uniform intensity either visually or by GenePix analysis. The two intensity values for each clone were quantified and plotted to assess the intra-array reproducibility.

A total of 192 scFvs from each specific antigen selection were recovered from the 3^rd ^and 4^th ^round of selection, purified from the periplasmic fraction of HB2151 *E. coli *cells and directly printed in duplicate onto FAST nitrocellulose slides (Figure 2A)[40-42]. Although some scFv expression differences were observed among the different clones, a correct printing pattern was detected, indicating adequate printing conditions (Figure 2B). The amount of scFv printed in the array was determined by printing serial 1:10 dilutions of previously reported anti-gastrin TA4 scFv at 0.5 μg/ml to construct a regression line [40]. Then, we used the median of the fluorescence signal of the scFvs in the regression line to get the amount of scFvs printed in the array. Approximately 12 pg of antibodies were printed in each spot of the array. The quality of the scFv antibody microarray was confirmed by the absence of cross contamination between successive spots. All positive controls as well as buffer spots showed the correct reactivity. The intra-assay reproducibility was assessed by comparing the results between the two replicas printed within the same chip for each clone and gave an R^2 ^= 0.9311 (Figure 2C).

### Evaluation of specific scFvs in microarray format

**Figure 3:**
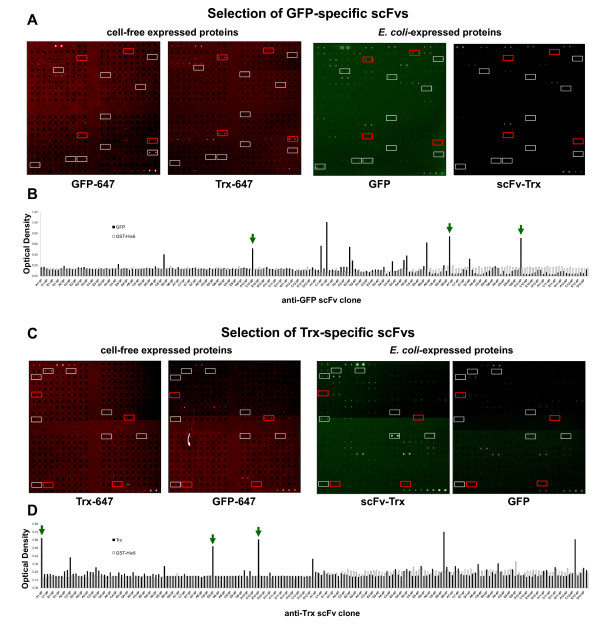
**Screening of specific scFvs using a microarray format**. Cell free-expressed proteins were labeled with 647 AlexaFluor and incubated with GFP- and Trx-specific scFvs microarrays to identify highly specific scFv binders. *E. coli-*expressed proteins were used as a control. A) Selection of GFP-specific scFvs. Left, performance of the microarray with cell free-expressed 647-labeled GFP or Trx as control. Right, performance of the microarray with *E. coli *expressed proteins followed by a polyclonal anti-GFP or a monoclonal anti-Flag and by AlexaFluor 555 labeled antibodies gave a green fluorescent signal. White boxes: 10 scFv antibodies against GFP showing at least 3-fold higher fluorescence signal than the control Trx values. Red boxes: scFvs showing non-specific binding for GFP. B) Anti-GFP scFv clones were tested by ELISA using GFP and GST to compare the microarray technology with ELISA screening. C) Selection of Trx-specific scFvs. Left, performance of the microarray with cell free-expressed 647-labeled Trx or GFP as control. Right, performance of the microarray with *E. coli*-expressed proteins followed by a polyclonal anti-GFP antibody or a monoclonal anti-Flag and by AlexaFluor 555 labeled antibodies. White boxes: 8 reactive scFv antibodies detected by antibody microarrays against Trx showing at least 3-fold higher fluorescence signal than the control GFP values. Red boxes: scFvs showing non-specific binding for Trx. D) Anti-Trx scFv clones were tested by ELISA using Trx, GST-His6 tagged and BSA to compare the performance of the microarray technology to identify Trx scFv binders with ELISA screening. Green arrows indicate the top three scFvs that gave the highest microarray signal for GFP or Trx and were further validated by other immunological techniques.

Evaluation of specific scFvs against GFP and Trx was performed by using scFv antibody microarrays. Cell-free expressed and purified GFP and Trx proteins were directly labeled with 647Alexa Fluor at 1 μg/mL to be probed in the antibody microarrays (Figure 3). In our microarrays, 384 recombinant scFvs were simultaneously tested. The scFvs obtained against each other antigen were also used as controls for the selection of highly-specific scFvs that did not show cross-reactivity to other antigen (Figure 3A,C).

To test the reproducibility and selectivity of the system, we used also GFP and Trx proteins expressed in *E.coli*, followed by a fluorescent labelled antibody (Figure 3A,C). Although signal intensity was usually stronger and background fluorescence was lower for the *E. coli*-derived proteins, a similar reactivity pattern was observed for the cell-free Alexa647-labeled purified protein (red) or the *E. coli*-expressed GFP and Trx (green), indicating an adequate performance of the expression system and the microarray screening.

To check the effect of a potential scFv denaturation during printing and to demonstrate the utility of the screening by antibody microarrays, we tested all the scFv binders for each antigen by ELISA. A comparable number of scFv binders were found by ELISA and by antibody microarrays. By antibody microarrays, we found 10 GFP-specific scFvs with 3-fold higher signals than the negative controls (microarray incubated with Trx). By ELISA, we found 13 scFvs with at least 2-fold higher signals than the negative controls (Figure 3B), obtaining a 62% of coincidence (8 GFP-specific scFvs) between ELISA and antibody microarray. Regarding Trx scFv binders, we found by ELISA 8 scFvs with 2-fold higher signals than the negative controls (Figure 3D). By antibody microarrays (Figure 3C) we also found 8 Trx-specific scFvs with 3-fold higher signals than the negative controls (microarray incubated with GFP). Indeed, we were able to detect 6 Trx scFv binders by both techniques, with a 75% coincidence between both techniques. Collectively, these data support the use of antibody microarray screening to identify highly-specific scFv antibodies as an alternative to conventional ELISA.

### Characterization of the selected scFvs

**Figure 4:**
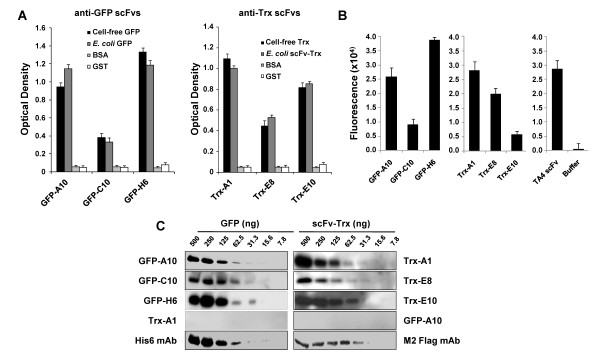
**Application of the scFvs in ELISA and WB**. A) Selected scFvs were tested by ELISA against the cell free-expressed proteins, *E. coli-*derived GFP and scFv-Trx 3xFlag proteins, using BSA and GST as negative controls. B) Intensity of the fluorescence signal of the GFP or Trx-specific scFvs in comparison to a scFv control signal or buffer obtained from the normalized data of the microarrays after incubation with 647Alexa Fluor labelled-GFP, 647Alexa Fluor labelled-Trx or anti c-myc followed by 555 AlexaFluor anti-mouse IgG, respectively. C) Different amounts of GFP or scFv-Trx were separated by SDS-PAGE and transferred to nitrocellulose membranes to determine the sensitivity of the anti-GFP and the anti-Trx scFvs (1:10 diluted) as primary antibodies. They were followed by an anti-c-myc tag and peroxidase-labeled anti-mouse IgG antibody, respectively. An anti-His6 mAb or an anti-M2 Flag mAb were used as positive controls to detect the target proteins.

To confirm the value of the scFvs obtained by antibody microarray screening, we characterized the three scFvs showing strongest reactivity against each *in vitro*-expressed antigen. These scFvs were tested by ELISA to compare their reactivity against the *in vitro*-expressed proteins and the *E. coli*-expressed proteins. The results were similar for the three anti-GFP scFvs (GFP-H6, GFP-C10 and GFP-A10) (Figure 4A, B). The two scFvs that showed strong recognition by antibody microarrays were also the best for ELISA. However, although Trx-A1 and Trx-E8 worked well in both assays (Figure 4A, B), Trx-E10 scFv showed a lower correlation between antibody microarrays and ELISA values.

The 6 scFvs selected (three per antigen) recognised their target proteins by western blot analysis, without cross-reactivity (Figure 4C). Indeed, most of the scFvs were able to detect as low as 31.3 ng of protein by western-blot, indicating the suitability of the scFvs for this technique. Indeed, Trx-A1 and Trx-E8 only showed 2-4 times lower sensitivity to Trx than M2 mAb to the Flag epitope (Figure 4C). Finally, the six scFvs were sequenced. All the scFvs-encoding DNA sequences were different (data not shown). The scFvs displayed significant variability not only in the CDRs but also in the framework as a consequence of the naïve origin of the Mehta libraries.

## Discussion

Antibodies are used to study protein expression and localization within a tissue, cell or organelle, protein-protein interactions or protein function [1,4,14,43,44]. Moreover, antibodies have multiple clinical uses for the diagnosis and treatment of diseases [5,20,21,45].

To study and characterize the human proteome, it is necessary to establish high-throughput methodologies for testing multiple antibodies against different proteins [10,12,13,46]. Here, we propose a new methodology to raise antibodies to any potential protein target: "antibodies on demand". This is a fast method to obtain recombinant human monoclonal antibodies against any antigen. Only 5 μg of protein were necessary to perform the whole procedure, since we took advantage of methodologies that require minimal amounts of protein: i) *in vitro *protein expression and purification, ii) antibody phage display and iii) scFv antibody microarray screening.

**Figure 5:**
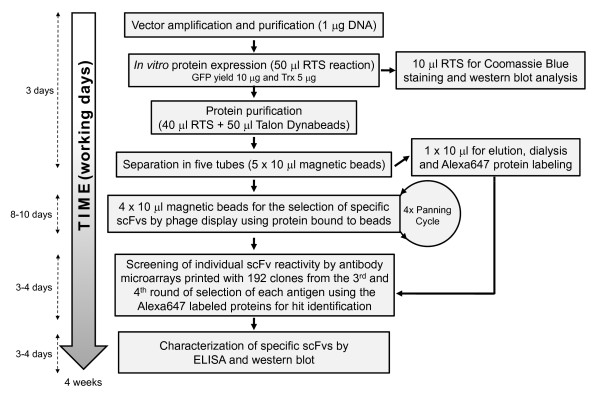
**Scheme of the full procedure**. Methodology and time schedule to produce antibodies on demand by using *in vitro *protein expression, phage display libraries and antibody microarrays.

This approach avoids the production of considerable amounts of protein necessary for both, the immunization and the screening steps, which usually are the bottlenecks for antibody development. In our hands, a dedicated person could produce antibodies in about 4 weeks, calculating 5 working days per week: i) 3 days for amplification, DNA purification and *in vitro *protein expression and purification by TALON™ Dynabeads^®^, ii) 8 days for three-four rounds of biopanning by phage display and *E. coli *amplification, iii) 3-4 days for printing and screening of scFv antibody microarrays and iv) 3-4 days for production and verification of the scFv results (Figure 5).

This methodology could be adapted to a high-throughput system. Indeed, all the techniques used in this report have been previously adapted to high-throughput assays [8,9,22,34,47,48]. One main advantage of our approach relies on the utilization of *in vitro *bacterial cell-free expression. Different reports support this expression strategy, because yields as low as 5 μg of protein were enough to get antibodies. Proteins with recovery yields below this amount could be scaled up *in vitro *as much as necessary to obtain the 5 μg of protein required for this approach.

Initially, cell-free expression was restricted by several limitations, especially for the expression of transmembrane proteins or proteins rich in disulfide bonds. However, advances in the *in vitro *technology have minimized these limitations by including several components, like canine pancreatic microsomal membranes, different DTT ratios and protein disulfide isomerase for the correct folding and production of proteins presenting disulfide bonds or different detergents and lipid bilayers for the expression of membrane proteins [25,26,49-52].

The microarray format for antibody screening shows different advantages: i) consumes minimal amounts of proteins, ii) is very sensitive, iii) improves the specificity, and iv) is suitable for high-throughput screening of antibodies. At the same time that we identified a recombinant antibody to a particular protein, a global-binding profile is simultaneously generated because different antibodies produced against different proteins are printed in the same array, permitting the selection of only highly specific, non cross-reactive antibodies. With our microarray settings, we could print up to 4800 spots in a high-density antibody format per slide. Since, only 192 scFvs were tested in duplicate per antigen, we could significantly increase the multiplexing of this methodology by testing antibodies against 12 targets simultaneously in the same slide.

Moreover, the use of recombinant antibodies in microarray format did not alter significantly scFvs binding properties after printing. It has been described that only a fraction of antibodies work well after printing on the surface of microarrays, due to the loss of activity by denaturation or degradation during printing and array storage. To explore the fact that we could have been losing 60-80% of total scFvs due to denaturation in our antibody microarrays [5,53], we decided to test all the scFvs by ELISA to compare the performance of the microarray and verify the usefulness of antibody microarrays for identification of scFv binders. Interestingly, we were able to identify a similar number of scFv binders by both screenings with a coincidence of 62 and 75% for GFP and Trx, respectively. These data support the utilization of antibody microarrays for the screening step, while consuming only 0.5 μg of 647 AlexaFluor protein.

Remarkably, scFvs were prepared by using a naïve scFv phage display library displaying multiple scaffolds (frameworks), avoiding the use of scFv antibody libraries specifically designed for antibody microarrays, which usually possess one fixed scaffold [48].

## Conclusion

We have developed a new and fast method to produce scFv antibodies by using only 5 μg of cell-free expressed protein and a naive scFv antibody library in combination with an antibody microarray format for screening. The scFvs were useful for different applications (ELISA, antibody microarrays and western blot). This antibody production technology could be especially applicable to "difficult-to-express" or toxic proteins, or when the amount of available protein might be a limiting step.

## Methods

### *In vitro *protein expression, purification and characterization

Plasmids pET32b-Trx or pIVEX-GFP were used in the cell free transcription/translation system to produce Trx or GFP, respectively. Plasmid DNA (1 μg) was ethanol precipitated and used as template for *in vitro *transcription/translation using the Rapid Translation System^® ^(RTS) kit (Roche Applied System). RTS reactions containing DNA template in 50 μL of solution (12 μL *E.coli *lysate, 10 μL reaction mix, 12 μL amino acids, 1 μL methionine, 5 μL reconstitution buffer and 1 μg of DNA in 10 μL RNAse-free water) were incubated at 30°C for 6 h at 300 rpm in a Thermomixer (Eppendorf). 10 μl RTS reaction for GFP and Trx was exclusively used to analyze the quality of the expressed and purified proteins with TALON™ Dynabeads^® ^by Coomassie Blue staining of 10% SDS-PAGE gels or, alternatively, by immunoblotting using an anti-His6-tag antibody (0.3 μg/ml, Roche). One RTS reaction was used to determine the enrichment of the biopanning procedure and to verify the scFv binding by ELISA.

BSA (Sigma), *E. coli-*expressed GFP-His6 (GE) and an irrelevant scFv fused to Trx (3xFlag, His6-tagged) were used as control proteins in this study. GST-His6 and scFv-Trx (3xFlag, His6) proteins were produced in *E. coli *according to established procedures [54]. Briefly, pET41b (Novagen) containing GST-cDNA and pSANG10-3F containing the cDNA encoding the scFv-Trx were used to transform BL21 (DE3) *E. coli *cells. His6-tagged proteins were then expressed and purified by affinity chromatography on a HiTrap chelating column (GE Healthcare).

### Selection of protein-specific scFvs from phagemid libraries

Human scFv Mehta libraries (a kind gift of Wayne Marasco, Dana-Farber Cancer Center. USA) were used for scFv production [55,56]. Mehta scFvs contain a c-myc tag for detection and purification. Phage display selections were performed using the proteins attached to TALON Dynabeads. Briefly, cell-free expressed proteins were attached to TALON™ Dynabeads through the His6 tag by incubating 40 μL of the RTS reaction with 50 μL of magnetic beads. Five equal aliquots were prepared, four for the rounds of selection and one for direct labelling of the proteins.

After exhaustive washing with PBS, magnetic beads were blocked with 3% BSA in PBS (PBS-BSA) for 1 h at room temperature. After washing, 100 μl of Mehta I and II libraries, diluted 1:10 in PBS-BSA, pre-incubated with magnetic beads to remove non-specific binders, were added to the solution and incubated for 2 h at room temperature. We performed four rounds of biopanning to get specific scFvs against GFP and Trx. Each round of selection included a negative subtraction of the phages against empty TALON™ Dynabeads^®^. Beads were washed 5 times with PBS-0.1% Tween during 2 min at room temperature and 40 rpm in a lab roller. Prior to elution a final washing step with PBS was performed to remove detergent from the media. Then, phages were eluted with 100 μL of 0.1M glycine, pH 2.7 during 10 min at room temperature in a lab roller at 40 rpm. Finally, phages were neutralized with 20 μL of 1M Tris, pH 9.

The amplification of the phages during the four rounds of biopanning was performed essentially as described by Barbas et al. [11] with minor modifications. Briefly, 100 μL of eluted phages from the first round of selection or 50 μL from the second and successive rounds of selection were used to infect 2 mL exponential phase XL1 Blue *E. coli *cells during 15 min at room temperature. Then, 6 mL of SB medium (10 g MOPS, 30 g of tryptone, 20 g of yeast extract in 1 L of water at pH 7.0) containing 1.6 μL of 100 mg/mL carbenicillin (Sigma) and 12 μL of 5 mg/mL tetracycline were added to the cell culture and further incubated at 37°C for 1 h at 250 rpm. Then, 2.4 μL of 100 mg/mL carbenicillin (Sigma) were added to the cell culture for an additional 1 h at 250 rpm at 37°C. Then, 1 mL VCSM13 helper phage (10^12 ^to 10^13 ^pfu) was added to the 8-mL cell culture together with 91 mL of prewarmed SB medium containing 46 μL of 100 mg/mL carbenicillin and 184 μL of 5 mg/ml tetracycline. The media was further incubated at 300 rpm for 2 h at 37°C. Finally, 140 μL of 50 mg/mL kanamycin was added to the medium and the solution was further incubated overnight at 37°C.

The day after, the culture was centrifuged at 4000 rpm for 15 min at 4°C. Supernatant containing phage suspension was mixed with 4 g of PEG-8000 (Sigma) and 3 g of NaCl (Merck) until complete dissolution and, then, was kept on ice for 30 min. Then, phages were centrifuged at 15000 g for 15 min and 4°C. Phages were resuspended in 2 mL of 1% BSA (Sigma) in PBS. Then, 100 μL out of 2 mL phage solution were directly used in subsequent rounds of biopanning as described above.

### Production of human scFv microarrays

Ninety six colonies were randomly picked from the 3^rd ^and 4^th ^round of biopanning against GFP and Trx. Individual colonies were added to 96-well plates containing 200 μL of 2xTY, 100 μg/ml ampicillin and 1% glucose and grown overnight at 37°C. Next day, cultures were diluted 1:100 in 96-well plates containing 200 μL of 2xTY, 100 μg/ml ampicillin and 0.1% glucose. IPTG was added at 1 mM final concentration and cultures were incubated overnight at 30°C. For scFv purification, cells were spun down, resuspended in 100 μl TES (10 mM Tris-HCl pH 8.0, 0.1 mM EDTA, 150 mM NaCl, 20% sucrose) and kept on ice for 30 min. The periplasmic fraction was obtained by centrifugation at 1800 rpm for 10 min [41,57]. Then, scFv-containing periplasms were diluted 1:2 in PBS, 0.1% Tween 20 (PBST) and directly arrayed in duplicate onto FAST slides (Whatman). Anti-T7 mouse monoclonal antibody (0.1 mg/mL, Novagen) and 1:10 fold dilutions starting at 0.1 mg/mL of TA4 scFv, which contains a c-myc tag at the C-terminus [40], were used as positive controls and PBST as negative control.

FAST nitrocellulose slides (Schleicher & Schuell, Whatman) were printed at 20°C and 45% of humidity using a microarrayer (Omnigrid, GeneMachines) with 4 micro-spotting 70 μm diameter stealth pins with reservoir (TeleChem). Pins were fixed to dip once and pre-spot 10 times before printing approximately 3 nL of solution per spot. The separation between dots was 350 μm. Slides were kept overnight inside the arrayer at 20°C and 75% of humidity for immobilizing the antibodies. Nitrocellulose slides were stored at -20°C until use.

### scFv microarray processing and data acquisition

Cell-free expressed GFP and Trx were labelled with a 647Alexa Fluor "Microscale Protein Labelling" kit (Invitrogen). Proteins attached to TALON™ Dynabeads^® ^were eluted with 50 mM phosphate buffer, 0.3M NaCl, 150 mM imidazole pH 8.0, and extensively dialysed against PBS. Purified proteins were lyophilised to concentrate before labelling.

Microarray slides were blocked with 4% skimmed milk in PBS (MPBS) for 1 h at room temperature. Then, either cell free AlexaFluor 647-labelled GFP or Trx (1 μg/mL), *E. coli-*expressed GFP or Trx (1 μg/mL) or anti-c-myc antibody (1 μg/mL, Roche) in 4% MPBS were added for 1 h at room temperature and incubated in SecureSeal hybridization chambers (Grace Bio-Labs). The microarrays were washed three times with PBST during 10 min. For detecting AlexaFluor 647-GFP or Trx, microarrays were air dried and directly scanned as described below. For detecting bound GFP and Trx proteins (or controls GFP-His6 and scFv-Trx-3xFlag), slides were incubated with rabbit polyclonal anti-GFP (0.2 μg/mL, Abcam) or monoclonal anti-Flag (10 μg/mL, Sigma) for 1 h, washed and incubated with Alexa Fluor 555-labeled goat anti-rabbit IgG (1μg/mL, Invitrogen) or Alexa Fluor 555-labeled goat anti-mouse IgG (1μg/mL, Invitrogen), respectively, for 1 h in the dark. For detecting c-myc antibody, slides were incubated with Alexa Fluor 555-labeled goat anti-mouse IgG antibodies (1 μg/mL). After three 10 min washes with PBST, microarrays were air dried and scanned with the ScanArray™ 5000 (Packard BioChip Technologies) using 635 nm and 532 nm lasers for Alexa 647 and Alexa 555, respectively. The Genepix Pro 4.0 image analysis software was used for quantification and analysis of the results.

### Sequence analysis

Phagemide DNAs from individual scFv positive colonies were amplified by PCR with the primers: pELB_forward, 5'-CATAATGAAATACCTATTGCCTA-3' and c-myc_reverse, 5'-CTTATTAGCGTTTGCCATT-3 [55]. Briefly, the cDNA encoding the scFvs was amplified using an initial denaturation step at 94°C for 2 min, followed by 30 cycles of 1 min at 94°C, 1 min at 55°C and 1 min at 72°C and a final step of 7 min at 72°C. Exonuclease I (USB) and shrimp alkaline phosphatase (USB) were added to the PCR products to remove any contaminant. Sequencing was carried out in an ABI7002 DNA sequencer (Applied Biosystems).

### ELISA and Western Blot analysis

For ELISA, flexible microtiter plates (Falcon, BD Biosciences) were coated overnight with 0.3 μg/well of the cell-free purified GFP or Trx. BSA or GST were used as negative controls. After washing three times with PBS, plates were blocked with 2% MPBS for 2 h at room temperature. Then, either different dilutions of the phage suspension or the periplasmic fractions were tested in the presence of 2% MPBS for 2 h at 37°C. After washing, peroxidase-labelled anti-M13 (1:5000 dilution in 2% MPBS) or anti-c-myc (1 μg/mL, Roche) to detect the phages or scFvs, respectively, were added for 1 h at 37°C. After washing, peroxidase reaction was developed with 3,3',5,5'-tetramethylbenzidine (TMB) substrate (Sigma). The reaction was stopped with 1M H_2_SO_4 _and the absorption measured at 450 nm.

For western blot analysis, proteins were transferred to nitrocellulose membranes. GFP and Trx were detected by using peroxidase-conjugated anti-His6 (0.3 μg/ml, Roche). Alternatively, GFP-His6 or scFv-Trx control proteins were detected with the corresponding scFvs at 100 ng/mL concentration. The signal was developed using an anti-c-myc tag (1 μg/mL, Roche) followed by peroxidase-conjugated anti-mouse IgG (0.2 μg/mL, Sigma). All the incubations were for 1 h at 37°C. ECL reagent was used for final detection.

Moreover, we have generated the fact sheets reporting the Minimum Information

About a Protein Affinity Reagent (MIAPAR), where we have included all the procedures and data reported for the scFvs produced in this study against GFP (Additional File 1) and Trx (Additional File 2).

## List of Abbreviations

scFvs: single-chain variable fragments; GFP: Green Fluorescent Protein; mAbs: monoclonal antibodies; MMP7: matrix metalloproteinase-7; pAbs: polyclonal antibodies; Trx: Thioredoxin.

## Authors' contributions

IB, RB and AP carried out the experimental work. IB and RB drafted the manuscript. IC conceived the study, supervised the experiments and wrote the manuscript. All authors read and approved the final manuscript.

## Supplementary Material

Additional file 1 **MIAPAR-compliant document for human anti-GFP scFvs**. The file includes the MIAPAR-compliant document presenting all the information about the production of human anti-GFP scFvs described in the manuscript. Click here for file

Additional file 2 **MIAPAR-compliant document for human anti-Trx scFvs**. The file includes the MIAPAR-compliant document presenting all the information about the production of human anti-Trx scFvs described in the manuscript. Click here for file
