# Supplementary material for: Antibodies on demand: a fast method for the production of human scFvs with minimal amounts of antigen
Source: BMC Biotechnol. 2011 Jun 2;11:61. doi: 10.1186/1472-6750-11-61 (PMC3125328; doi:10.1186/1472-6750-11-61)
Supplement: Additional file 1 — MIAPAR-compliant document for human anti-GFP scFvs. The file includes the MIAPAR-compliant document presenting all the information about the production of human anti-GFP scFvs described in the manuscript. [file 1472-6750-11-61-S1.PDF]

Additional File 1. A MIAPAR-compliant document presenting information described in the manuscript entitled "Antibodies on demand: a fast method for the production of human scFvs with minimal amounts of antigen" by Babel *et al*, 2011.

1. Header

**1.1. Responsible person or role**	
**1.1.1 Contact Person**	Ignacio Casal
**1.1.2. Organization**	Functional Proteomics Laboratory, Centro de Investigaciones Biológicas, CSIC. c/ Ramiro de Maeztu, 9. 28040 Madrid. Spain
**1.1.3. Contact E-mail**	icasal@cib.csic.es

2. Molecular entities

**2.1. Target**		
**2.1.1. Description**		Green Fluorescent Protein (GFP)<br>UniProtKB id: P42212
**2.1.2. Production**		*In vitro* protein expression using Rapid Translation System (RTS) kit from Roche Applied System
**2.1.3. Molecular Characterization**		Mass fingerprinting
**2.2. Affinity Reagent**		
**2.2.1. Description**	**2.2.1.1. Affinity Reagent Identifier**	Monoclonal recombinant antibodies to GFP.<br>*Clone name*: GFP-A10, GFP-C10 and GFP-H6<br>See (Babel et al, 2011)
	**2.2.1.2 Affinity reagent class**	Single Chain Variable Fragment (scFv)
**2.2.2 Production**	**2.2.2.1 Host organism**	Phage Display human Mehta I and II scFv antibody libraries
	**2.2.2.2 Affinity reagent synthesis and purification**	Phage Display Technology to obtain scFvs against GFP. *See Detailed steps below.*

2.2.2.2 Affinity reagent synthesis and purification

Step name	Materials	Reagents	Methods
In vitro protein expression	Rapid Translation System kit (Roche Applied System)<br><br>Thermomixer (Eppendorf)<br><br>Talon-Dynabeads (Invitrogen)	pIVEX-GFP plasmid	Cell-free expressed GFP was obtained after incubating plasmid DNA pIVEX-GFP (1μg) in 50 μl of RTS reaction solution during 6 h at 30 ºC and 600 rpm in a Thermomixer.<br><br>40 μL of the RTS reaction were incubated with 50 μL of magnetic beads. After extensive washing with PBS, attached GFP to Talon Dynabeads through its His6 tag was splitted in 4 tubes to perform 4 rounds of selection of scFvs by Phage Display.
Phage Display antibody libraries biopanning	Mehta I an II human phage display antibody libraries from Dana-Farber Cancer Center (Wayne Marasco, PhD, MD)<br><br>GFP (10 μg) cell-free expressed<br><br>Talon-Dynabeads (Invitrogen)	Phage Display human antibody libraries	Biopanning procedure was performed using GFP attached to Talon Dynabeads as described by Babel et al. (2011).
Labeling of cell-free expressed GFP	647Alexa Fluor “Microscale Protein Labelling” kit (Invitrogen)	Cell-free expressed GFP	10 μL of the RTS reaction were incubated with 10 μL of magnetic beads, washed extensively with PBS and eluted with imidazole. After extensive dyalisis against PBS, GFP was lyophilised and labeled with 647Alexa Fluor “Microscale Protein Labelling” kit according to the suggestions of the manufacturer.

scFv expression and purification	Centrifuge<br>Incubator	2xTY<br>IPTG<br>Tris, EDTA, NaCl, Sucrose	Individual selected clones were added to a suitable volume of 2xTY, 100 µg/ml ampicillin and 1 % glucose and grown overnight at 37ºC. Next day, cultures were diluted 1:100 in 2xTY, 100 µg/ml ampicillin and 0.1 % glucose. IPTG was added at 1 mM final concentration when Optical Density reached 0.9 at 600 nm. Cultures were incubated overnight at 30ºC. For scFv purification, cells were spun down, resuspended in TES (10 mM Tris-HCl pH 8.0, 0.1 mM EDTA, 150 mM NaCl, 20% sucrose) and kept on ice for 30 min. Then, 1.5 volumes of 1:5 TES was added to the cell solution, kept on ice for extra 30 min. The periplasmic fraction was, then, obtained by centrifugation at 10000 rpm for 10 min.
Evaluation of specific scFvs in microarray format	192 scFvs from the third and fourth round of biopanning against GFP expressed in HB2151 *E. coli* cells<br>Microarrayer (Omnigrid, GeneMachines)<br>Micro-spotting 70 µm diameter stealth pins with reservoir (TeleChem).<br>ScanArray™ 5000 (Packard BioChip Technologies) and Genepix Pro 4.0 (Axon Laboratories)	PBS<br>Tween 20<br>Nitrocellulose Fast Slides (Whatman)<br>GFP cell-free expressed labeled with 647Alexa Fluor "Microscale Protein Labelling" kit (Invitrogen)<br>Non-fat milk	Microarray slides printed with the periplasmic fraction of 192 scFvs were blocked with 4% non-fat milk in PBS for 1 h at room temperature. Then, cell free AlexaFluor 647-labelled GFP (1µg/mL) was added for 1 h in 4% MPBS at room temperature. Microarrays were washed three times with PBST during 10 min. Microarrays were air dried and scanned with the ScanArray™ 5000 using 635 nm laser for Alexa 647. The Genepix Pro 4.0 image analysis software was used for quantification and analysis of the results and identification of the reactive scFvs.

3. Affinity Reagent / Target Interaction

3.1. Binding Description	
**3.1.1. Sensitivity**	The three anti-GFP scFvs detected 31 ng of GFP by western blotting analysis.
**3.1.2. Epitope recognized**	The epitopes of the antibodies in GFP were not determined.
**3.1.3. Binding Constant**	The binding constant for GFP was not determined.
**3.1.4. Selectivity**	The three scFvs anti-GFP specifically bound GFP but failed to recognize Trx, GST, BSA and polyHis proteins as revealed by ELISA, antibody microarrays and western blotting.

**3.1.5. Applications**	ELISA, Western blotting, Antibody microarrays	
**3.2. Sensitivity to GFP**		
**3.2.1. Experimental Method**	ELISA.<br>*Materials:*<br>GFP (P42212). Purified scFvs (100 ng) diluted with 2% non-fat milk (w/v) in PBS 1x pH 7.3<br>*Reagents :*<br>PBS 1x buffer pH 7.3<br>Peroxidase anti-c-myc (1 μg/mL, Roche)<br>Blocking solution 2% non-fat millk<br>TMB as peroxidase substrate. 1M HCl as stopper solution.<br>Absorption measurement at 450 nm	
**3.2.2. Target Identity**	GFP (P42212)	
**3.2.3. Target State**	Purified	
**3.2.4. Results**	The three scFvs anti-GFP detected 100 ng of purified GFP. The interaction was not affected by the presence of non-fat milk 2%.	
**3.3. Selectivity**		
**3.3.1. Experimental Method**	ELISA<br><br>scFv anti-GFP C10, scFv anti-GFP A10 and scFv anti-GFP H6<br>*Materials :*<br>GFP and control proteins	Antibody microarray<br><br>scFv anti-GFP C10, scFv anti-GFP A10 and scFv anti-GFP H6<br>Materials:<br>GFP and control proteins<br>Nitrocellulose Fast Slides microarrays printed with scFvs
**3.3.2. Target Identity**	GFP	GFP
**3.3.3. Target State**	Natural	Natural
**3.3.4. Results**	Selected scFvs specifically bound GFP but did not recognize BSA, Trx, GST and polyHis proteins.	Selected scFvs specifically bound GFP but did not recognize BSA, Trx, GST and polyHis proteins.

4. Affinity Reagent Applications

4.1. Immunoblotting study (Babel et al., 2011)

**4.1.1. Experimental Method**	Western blotting<br>scFv anti-GFP C10, scFv anti-GFP A10 and scFv anti-GFP H6<br>*Materials:*<br>GFP<br>scFv anti-GFP (100 ng/mL) in PBS, non-fat milk 3%<br>anti-c myc tag monoclonal antibody (1 μg/mL, Roche)<br>Peroxidase-conjugated anti-mouse IgG (0.2 μg/mL, Sigma)<br>*Reagents:*<br>Non-fat milk<br>PBS
**4.1.2. Target Identity**	Different amounts of GFP ranging from 500 to 7.8 ng.
**4.1.3. Target State**	Denatured
**4.1.4. Results**	The scFvs were able to recognise as low as 31 ng of denatured GFP. GFP-C10 scFv recognized 15.6 ng of GFP.
